# Differential association of lead on length by zinc status in two-year old Mexican children

**DOI:** 10.1186/s12940-015-0086-8

**Published:** 2015-12-30

**Authors:** Alejandra Cantoral, Martha M. Téllez-Rojo, Teresa Shamah Levy, Mauricio Hernández-Ávila, Lourdes Schnaas, Howard Hu, Karen E. Peterson, Adrienne S. Ettinger

**Affiliations:** Research Center for Nutrition and Health, National Institute of Public Health, Universidad No. 655 Colonia Santa María Ahuacatitlán, Cerrada Los Pinos y Caminera, C.P. 62100 Cuernavaca, Mor Mexico; National Institute of Public Health, Universidad No. 655, Colonia Santa María Ahuacatitlán, Cerrada Los Pinos y Caminera, C.P. 62100 Cuernavaca, Mor Mexico; National Institute of Perinatology, Monte Urales Sur 800, Lomas Virreyes, C.P. 11000 Mexico City, Mexico; Dalla Lana School of Public Health, University of Toronto, 155 College St, 6th floor, Toronto, ON M5T 3M7 Canada; Department of Nutritional Sciences, University of Michigan School of Public Health, 1415 Washington Heights, Ann Arbor, MI 48104 USA; Department of Nutrition, Harvard School of Public Health, 677 Huntington Avenue, Boston, MA 02215 USA

**Keywords:** Children, Lead, Length-for-age, Stature, Zinc status

## Abstract

**Background:**

Blood lead levels have decreased in Mexico since leaded fuel was banned in 1997, but other sources remain, including the use of lead-glazed ceramics for food storage and preparation. Zinc deficiency is present in almost 30 % of children aged 1–2 years. Previous studies have documented negative associations of both lead exposure and zinc deficiency with stature, but have not considered the joint effects. Given that the prevalence of stunting in pre-school aged children was 13.6 % in 2012, the aim of this study was to evaluate if the relationship between blood lead and child stature was modified by zinc status.

**Methods:**

Anthropometry, dietary energy intake, serum zinc and blood lead were measured in 291 children aged 24 months from an ongoing birth cohort study in Mexico City. Child stature was represented by recumbent length as appropriate for this age group. The association between blood lead (BPb) and length-for-age Z score (LAZ) was evaluated using a model stratified by zinc status measured by standard criteria and adjusted for: birth length, breastfeeding practices, energy intake, maternal height and education.

**Results:**

Median (IQR) BPb was: 0.17 (0.12–0.26) μmol/L and 17 % of the sample had zinc deficiency (<9.9 μmol/L). BPb was inversely associated with LAZ in the overall sample (β = −0.19, *p* = 0.02). In stratified models, this negative association was more than three times higher and statistically significant only in the zinc deficient group (β = −0.43, *p* = 0.04) compared to the zinc replete group (β = −0.12, *p* = 0.22) (BPb*zinc status, p-for-interaction = 0.04).

**Conclusions:**

Zinc adequacy is a key factor that may attenuate the negative association of lead on stature in young children.

## Background

Children’s blood lead (BPb) levels have declined worldwide following the removal of lead from gasoline. Leaded gasoline was completely phased out in Mexico by 1997 which brought about a significant reduction in population exposures and a subsequent reduction in BPb [1, 2]. However, other sources of lead exposure remain including lead-glazed ceramics used for food storage and preparation which is now a main source of exposure in the population [3, 4]. A recent report estimated that the geometric mean BPb in Mexican children is 0.31 μmol/L (6.5 μg/dL) [5] which is higher than the CDC reference level of 0.24 μmol/L (5 μg/dL) [6].

It has been documented that young children can absorb up to 59 % of ingested lead [7, 8]. Lead absorption is enhanced by diets low in calcium, iron and zinc and depends on the micronutrient status of the gastrointestinal lumen [9, 10]. The toxic effects of lead may also be modified by the availability of these essential elements [9, 11]. While lead and zinc interactions in humans are not as well defined as those between lead and calcium or iron, it has been shown experimentally that lead increases zinc excretion [12].

Zinc plays an important role as a necessary nutrient for growth by its participation in numerous enzyme systems [13]. Moderate zinc deficiency in pre-school aged children is common in developing countries and can delay linear growth [14, 15]. The 2006 Mexican National Health and Nutrition Survey documented a prevalence of zinc deficiency of almost 30 % in children aged 1–2 years [16]. Additionally, in 2012, 13.6 % of children below 5 years of age were shown to exhibit stunting (<2 standard deviations (SD) length/height-for-age Z score) [17].

The negative association of BPb with child stature has been documented in cross-sectional analyses [18–21] and cohort studies [22–24]. It is unclear whether this association differs according to nutritional status, but this could be relevant in a country with high occurrence of stunting, moderate zinc deficiency, and concurrent exposures to lead. The aim of this study was to assess the association between blood lead and child stature for age and sex (using length-for-age Z score (LAZ)) and to investigate if this association differed by zinc status in a cohort of Mexican children at 24 months of age.

## Methods

This cross-sectional study was nested in the second birth cohort (1997–2000) of the *Early Life Exposure in Mexico to Environmental Toxicants* (ELEMENT) Project. Briefly, women were enrolled during pregnancy in a larger study examining prenatal lead biomarkers and child neurodevelopment in Mexico City [25, 26]. Children were followed from birth through 60 months of age.

As the parent study for this project did not include measures of serum zinc in all the visits, we only evaluated children who attended at the 24-month visit, when a subsample of children had both serum zinc and blood lead available. The study visit was completed at the National Institute of Perinatology Isidro Espinosa de los Reyes (*INPer* by its abbreviation in Spanish) between 2001 and 2003, when the children were 24 (±1) months of age.

From the 443 participants who attended the 24-month visit, we excluded: infants with very low birth weight (<1500 g) (*n* = 2, 0.5 %) and premature birth (<37 weeks gestation) (*n* = 19, 4.2 %) both of which are associated with decreased stature [27]. In addition, there were 131 children (29.5 %) without zinc serum determination, thus giving a final sample of 291. Besides the first two variables, the participants in this analysis and the rest of the cohort are similar with respect to sociodemographic characteristics (data not shown).

During a morning visit (between 9:00 and 11:00 AM), non-fasting venous blood samples were drawn from children and anthropometric measures were obtained by trained personal. Whole blood samples were centrifuged and serum was isolated at the *INPer* laboratory, where zinc concentrations were determined through flame atomic absorption spectrometry (FAAS), the technique deemed most suitable for this procedure [28]. Serum zinc values were used as the criterion measure and children were classified as having zinc deficiency with values <9.9 μmol/L (65 μg/dL) [29]. A sample of blood was collected in a free-metal tube and BPb was determined by graphite furnace atomic absorption spectrophotometry (Perkin-Elmer 3000, Chelmsford, MA) at the American British Cowdray Hospital Laboratory using previously described methods [30].

Recumbent length was measured using an infantometer (Seca Infantometer 417, Seca North America, Chino, CA USA) to the nearest millimeter according to standard anthropometric methodology [31] by trained and standardized personnel. Up to 2 years of age, measurements of linear growth are based on recumbent length with change to measurement of standing height (stature) recommended after 2 years of age [32]. Weight was measured using a pediatric scale (Health o meter 533KL, Sunbeam Products, Inc. Boca Raton, FL USA) to the nearest 100 g. WHO Anthro (version 3.2.2, January 2011, World Health Organization, Geneva, Switzerland) software was used to estimate the LAZ and stunting was defined as LAZ <2 SD [33].

Maternal characteristics, including age, height, and education were recorded in a questionnaire administered by a trained social worker; mother’s height was measured at third trimester of pregnancy; education was asked as a proxy of socioeconomic level and was recorded as the total number of years studied. Birth characteristics, such as child’s sex, weight and length were obtained from the birth certificate. Breastfeeding practices (exclusive, partial, none) and total number of months of breastfeeding (any type) were asked of the mother at 1, 3, 6, and 12 months postpartum. The child’s daily energy intake at 24 months of age was assessed using a validated semi-quantitative food frequency questionnaire [34] administered to the mother.

### Ethics, consent, and permissions

This study was evaluated and approved by the Research, Ethics and Biosafety Committees of the National Institute of Public Health of Mexico (*INSP* by its abbreviation in Spanish) and all participating institutions. Mothers of the cohort-enrolled children were apprised of the study procedures and objectives, and signed an informed consent prior to participation.

### Statistical analysis

Descriptive statistics were computed depending on the nature of the variable and were compared stratified by zinc status. For continuous variables, means and SD were computed; T-tests were used to compare groups with normally-distributed variables and a Wilcoxon rank sum test for skewed variables. For categorical variables, median and interquartile range and proportions were computed and compared using the two-sided Fisher’s Exact test.

Multivariate linear models were used to estimate the overall association between lead and LAZ and also stratified by zinc status. When the models were run with untransformed BPb, residuals had non-normal distribution; thus, we included log-transformed BPb to normalize the residuals and improve model fit. Models were adjusted for measured variables that are recognized as predictors of child stature/length, including: birthlength (cm), breastfeeding (total months), energy intake (Kcal/day), maternal height (cm) and years of education [35–37]. We did not include prenatal lead exposure or prenatal zinc deficiency because these variables may directly affect birth characteristics (weight, length and gestational age) [30]. We chose to restrict our analyses to include only infants with adequate birth weight and normal gestational age. In addition, the percentage of mothers in the cohort who smoked cigarettes or drank alcohol during pregnancy is less than 2 %; hence, we did not include those variables. To test for effect modification, we created an interaction term between lead and zinc status (lead*zinc status) that was included with the main lead and zinc status terms and covariates from the final model (data not shown).

All analyses were performed using STATA® version 11.0 (Copyright 1985–2009, StataCorp LP College Station, Texas USA).

## Results

Table 1 presents the descriptive characteristics for the 291 children (51.5 % male). Zinc deficiency was present in 17 % of children, but none of the characteristics were significantly different by zinc status. Mean weight and length at birth were in the normal range for gestational age based on study exclusion criteria. At 24 months of age, seven participants (2.4 %) presented stunting. Median (IQR) BPb was: 0.17 (0.12–0.26) μmol/L; 85 children (29 %) had BPb >0.24 μmol/L (5 μg/dL) and 14 (5 %) had BPb >0.48 μmol/L (10 μg/dL), representing the first one, the current CDC reference range for children, and the second one the former CDC reference for children and current reference for children in the Mexican Norm (NOM-199-SSA1-2000) [6]. Serum zinc ranged from 6.5 to 25.0 μmol/L with mean concentrations of 12.9 and 9.0 μmol/L, respectively, in the zinc replete and zinc deficient groups.

**Table 1:** Characteristics of the study population at 24 months of age, overall and stratified by zinc status

	Overall (*N* = 291)	Zinc Replete (*N* = 241)	Zinc Deficient (*N* = 50)	
	Mean ± SD (or *N* (%))			*p*-value*
Sex (male)	150 (51.5 %)	123 (51 %)	27 (54 %)	0.7
Birthweight (kg)	3.1 ± 0.4	3.1 ± 0.4	3.1 ± 0.4	0.6
Birthlength (cm)	50.1 ± 1.9	50.1 ± 1.9	50 ± 1.5	0.4
Gestational Age (weeks)	39.0 ± 1.1	39.0 ± 1.1	39 ± 0.9	0.7
Age at anthropometry (months)	24.5 ± 0.2	24.5 ± 0.2	24.5 ± 0.3	0.8
Weight (kg)	11.9 ± 1.5	11.9 ± 1.5	11.6 ± 1.2	0.1
Length (cm)	86.3 ± 2.9	86.4 ± 2.9	86.1 ± 2.8	0.5
Length-for-Age Z score	−0.02 ± 0.9	−0.01 ± 0.9	−0.1 ± 0.9	0.5
Stunting (<2 SD LAZ)	7 (2.4 %)	6 (2.5 %)	1 (2.0 %)	0.6
Serum Zinc (μmol/L)	12.2 ± 2.5	12.9 ± 2.2	9.0 ± 0.9	<0.01
Blood Lead (μmol/L)^a^	0.17 (0.12–0.26)	0.17 (0.12–0.27)	0.15 (0.12–0.23)	0.2
Blood Lead >0.24 μmol/L	85 (29 %)	74 (31 %)	11 (22 %)	0.1
Blood Lead >0.48 μmol/L	14 (5 %)	13 (5 %)	1 (2 %)	0.3
Energy Intake (Kcal/d)	1656.3 ± 426.2	1672.0 ± 432.6	1590.0 ± 390.3	0.2
Breastfeeding (months)	8.6 ± 6.2	8.9 ± 6.3	7.9 ± 5.6	0.3
Maternal Height (cm)	155.0 ± 5.7	155.0 ± 5.6	154.7 ± 6.0	0.8
Maternal Education (years)	11.0 ± 2.9	11.0 ± 2.8	11.5 ± 3.3	0.3

*p-values for the comparison of the zinc replete and zinc deficient groups (T-tests were used for continuous and normally-distributed variables, Wilcoxon rank sum test for continuous and skewed variables, and two-sided Fisher’s Exact test for categorical variables)

^a^Median (interquartile range) and Wilcoxon test

In Table 2, models are presented for the entire sample and stratified by zinc status. Overall, we observed a negative association of lead (log units) on LAZ (β = −0.19, *p* = 0.02) which represents a 17 % reduction in LAZ for every 1-unit increase in blood lead. Birthlength and mother’s height were positively and significantly associated with LAZ in the overall sample. Among zinc replete children, lead was inversely associated with LAZ (β = −0.12, which represents an 11 % reduction in LAZ for every 1-unit increase in blood lead), but this association was not statistically significant (*p* = 0.22). In zinc replete children, the two covariates that significantly predicted LAZ at 24 months of age were birthlength (β = 0.13, *p* <0.01) and mother’s height (β = 0.04, *p* <0.01). For the group of children with zinc deficiency, none of the covariates significantly predicted LAZ and only lead (log units) negatively and significantly predicted LAZ (β = −0.43, *p* = 0.04) which represents a 35 % reduction in LAZ for every 1-unit increase in blood lead.

**Table 2:** Adjusted association of log blood lead (μmol/L) and covariates on length-for-age Z score (LAZ), overall and stratified by zinc status

	Overall (*N* = 291)			Zinc Replete (*N* = 241)			Zinc Deficient (*N* = 50)		
	β	SE	95 % CI	β	SE	95 % CI	β	SE	95 % CI
Log Blood Lead (μmol/L)	−0.19	0.08	−0.36, −0.03	−0.12	0.09	−0.30, 0.07	−0.43	0.20	−0.84, −0.02
Birthlength (cm)	0.10	0.03	0.05, 0.16	0.13	0.03	0.07, 0.18	−0.03	0.07	−0.18, 0.12
Energy Intake (1000 Kcal/d)	0.03	0.12	−0.26, 0.20	−0.11	0.13	−0.36, 0.13	−0.01	0.32	−0.65, 0.62
Breastfeeding (months)	−0.01	0.01	−0.03, 0.00	−0.01	0.01	−0.03, 0.01	−0.02	0.02	−0.06, 0.03
Maternal Height (cm)	0.04	0.01	0.02, 0.06	0.04	0.01	0.02, 0.06	0.02	0.02	−0.02, 0.06
Maternal Education (years)	−0.01	0.01	−0.03, 0.01	−0.02	0.01	−0.04, 0.00	0.04	0.04	−0.03, 0.11
Adjusted R-squared	0.15			0.17			0.16		

**Fig. 1:**
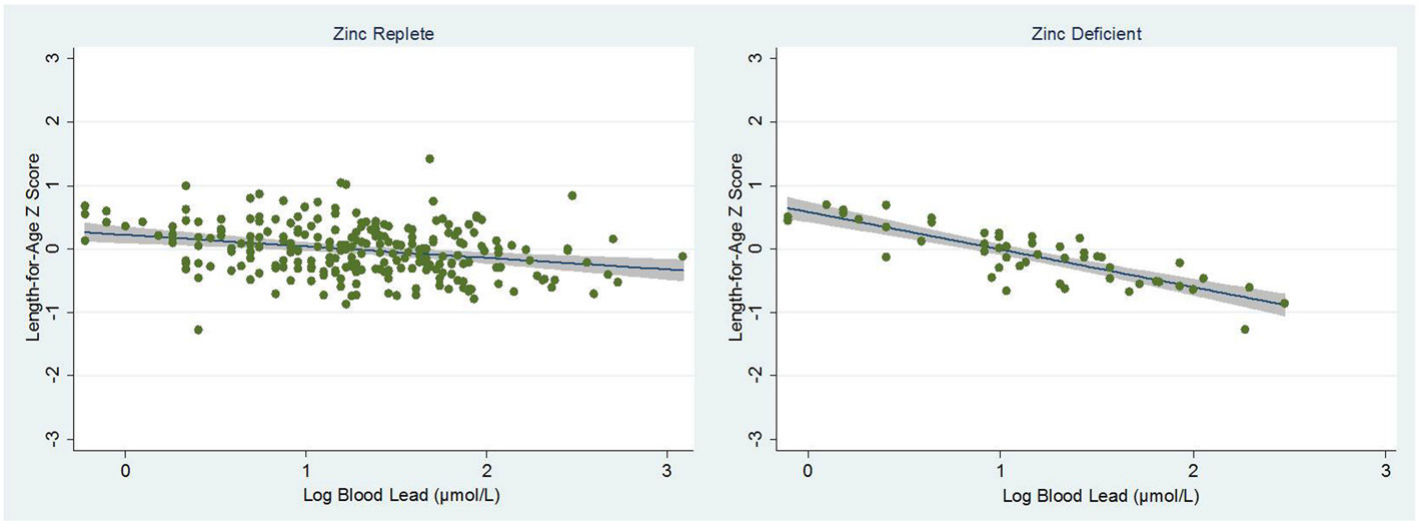
Observed and predicted values (and 95 % CI) of the regression of length-for-age Z score on log blood lead (μmol/L) by zinc status. (Observed data are represented by *circles*; predicted values are represented by *lines with gray shading* indicating the 95 % CI)

Predicted values and 95 % CI of the regression of LAZ on BPb (log units) according to zinc status are presented in Fig. 1. While the observed slopes for the two groups are both negative, the slope for the zinc deficient group is clearly greater than that of the replete group indicating that there is a stronger negative association of blood lead on length in children with zinc deficiency. In fact, the zinc-deficient β value represents a percentage reduction in LAZ with increasing blood lead that is more than three times that of the zinc-replete β value and the difference in β values is confirmed by the *p*-value of 0.04 for the BPb*Zn status interaction term.

## Discussion

We observed, and to our knowledge are presenting such results for the first time, that the association between lead and child’s length differs by zinc status. Our study confirms a previously reported negative association between lead and stature in young children. Even though the study sample had lower BPb than previous reports in Mexican children [38, 39], we found a negative association between log BPb and LAZ at 24 months of age in the overall sample (β = −0.19, 95 % CI: −0.36, −0.03) which represents a 17 % reduction in LAZ for every 1-unit increase in blood lead. This is consistent with previous reports of lead associated with decreased stature in Hispanic populations [20], in other cohorts [24], and with results from our study group [22].

When we stratified our models by zinc status, the negative association of BPb on LAZ was observed in both the zinc replete and zinc deficient groups; however, the association was only statistically significant in the zinc deficient group despite that group having a smaller sample size. A post-hoc analysis of the power related to the number of study subjects found that our study of 291 subjects had power of more than 70 % to detect the observed difference. In the zinc deficient children, the negative effect of BPb (β = −0.43, 95 % CI: −0.84, −0.02) representing a 35 % reduction in LAZ was more than three times higher than the negative effect in the zinc replete group (β = −0.12, 95 % CI: −0.30, 0.07) representing an 11 % reduction in LAZ (p-for-interaction = 0.04).

Lead is thought to negatively affect skeletal growth by altering osteoblast and osteoclast function, cell differentiation, reduction in plasma concentration of active vitamin D and by competing with calcium [40] which could be at least partly responsible for the effects of lead on stature. Zinc is important for growth due to its role in elongation and maintenance of bone at the levels of regulation of the hormonal axis and of signaling within the cellular elements of cartilage and bone [13]. Lead can substitute for zinc in various zinc-mediated processes and has been shown experimentally to increase zinc excretion [9]. Additionally, dietary zinc has been shown experimentally to reduce lead, as observed in a rat study where zinc supplementation reduced lead concentrations after three months of treatment [41].

These findings are important because malnutrition is a primary risk factor for growth restriction in children and, additionally, early-life environmental exposures appear to play a role. Specifically, zinc deficiency negatively affects children’s linear growth and increases the risk and severity of diarrhea, pneumonia and other infections. However, even in the absence of zinc deficiency, lead exposure also has negative effects on growth among zinc replete children. Also, subclinical deficiencies may be common especially in populations with low consumption of zinc-bioavailable foods or diets high in zinc inhibitors, such as high phytate diets common in Mexico, which inhibit zinc absorption, thus leaving individuals more susceptible to the toxic effects of lead [42].

Our study has several limitations including the cross-sectional analysis, a relatively small sample size, especially in the zinc deficient group, and the lack of information on other variables that affect stature such as father’s height and the number of acute infections experienced by the child. The children in this analysis were limited to full‐term infants without low birthweight or length which may be responsible for the low variability in LAZ. Also, our study participants are all from a relatively homogeneous, low‐to‐moderate income, urban population. We recognize that the FFQ is subject to measurement error, specifically the potential for recall bias, but we do not believe that this would be differential with respect to lead exposure or the outcome of interest. Additionally, we used objective serum measures of zinc status in this study. We did not control for maternal smoking or alcohol consumption due to the low prevalence of these behaviors reported in our study population. Even though we do not specify the type of breastfeeding practices here, we believe that our quantitative breastfeeding variable (total months over 12 months postpartum) appropriately estimates the effect of breastfeeding given that, in Mexico, the prevalence of exclusive breastfeeding is low during the first 6 months of life (only 25 % of children were exclusively breastfed according to the 2012 National Health and Nutrition Survey).

Our results suggest that zinc has important benefits, beyond those commonly recognized such as preventing infections, particularly in populations at risk for both dietary deficiencies and environmental exposures to toxic chemicals such as lead. In addition, individuals with genetic mutations or polymorphisms of genes regulating metal binding and transporter proteins, such as metallothionein and divalent metal transporter 1 (DMT1), may be particularly susceptible to metal nutrient interactions [13]. Zinc status is also a reflection of general nutritional status, so adequate zinc may only be one factor in nutritional status that helps ward off the negative effects of lead exposure [43]. Nonetheless, dietary zinc supplementation should be considered part of a broader effort to improve the overall nutritional status of children, especially during the first 2 years of life [44–46] and particularly among children who may face additional burdens such as lead exposure. Young children at risk for lead exposure should avoid sources of exposure and maintain adequate nutritional status particularly in areas where nutritional deficiencies are known to exist.

## Conclusions

Zinc adequacy is a key factor that may attenuate the negative association of lead on stature in young children. Based on these results, further research is needed before specific recommendations can be made.

## Abbreviations

BPb: Blood Lead
CDC: U.S. Centers for Disease Control and Prevention
ELEMENT: Early Life Exposure in Mexico to ENvironmental Toxicants
LAZ: Length-for-Age Z score
SD: Standard Deviation
WHO: World Health Organization
